# Supplementary material for: Rapid gene content turnover on the germline-restricted chromosome in songbirds
Source: Nat Commun. 2023 Jul 29;14:4579. doi: 10.1038/s41467-023-40308-8 (PMC10387091; doi:10.1038/s41467-023-40308-8)
Supplement: Supplementary file 1 — Supplementary Information [file 41467_2023_40308_MOESM1_ESM.pdf]

Rapid gene content turnover on the germline-restricted chromosome in songbirds

Supplementary Information

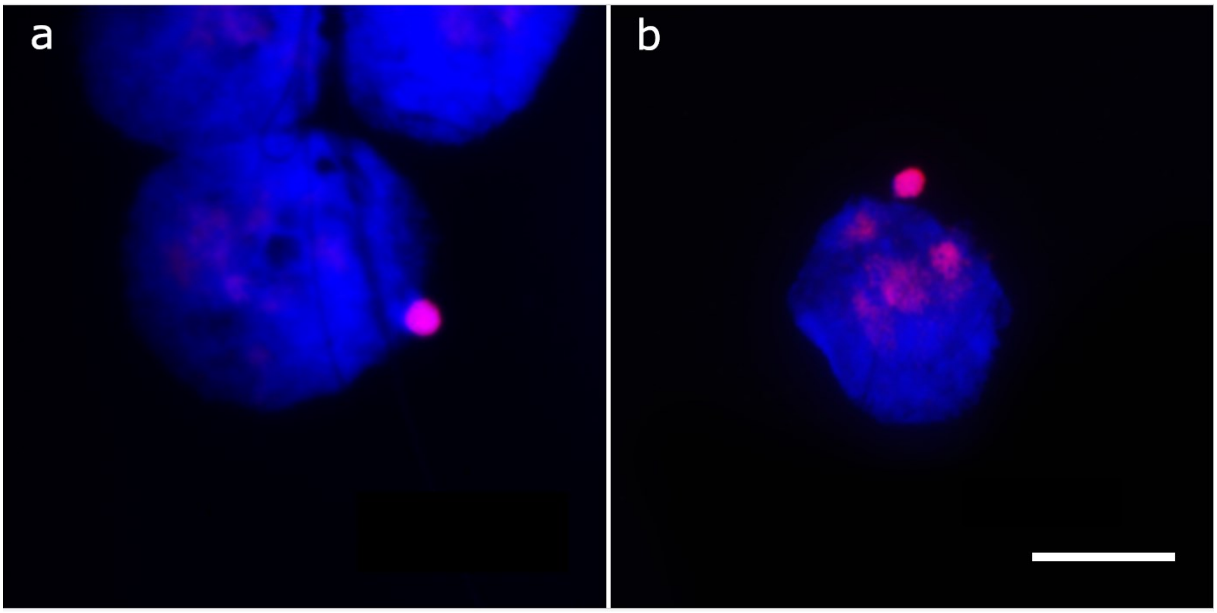

Supplementary Figure 1: Visualisation of the expelled micronucleus. Micronucleus containing the eliminated GRC next to secondary spermatocytes in *Luscinia megarhynchos* (**a**) and *L. luscinia* (**b**). Micronuclei are immunostained with H3K9me antibody (pink) and DNA is counterstained with DAPI (blue). For each species, the expelled micronucleus was observed five times in two different individuals. Scale bar represents 10 µm.

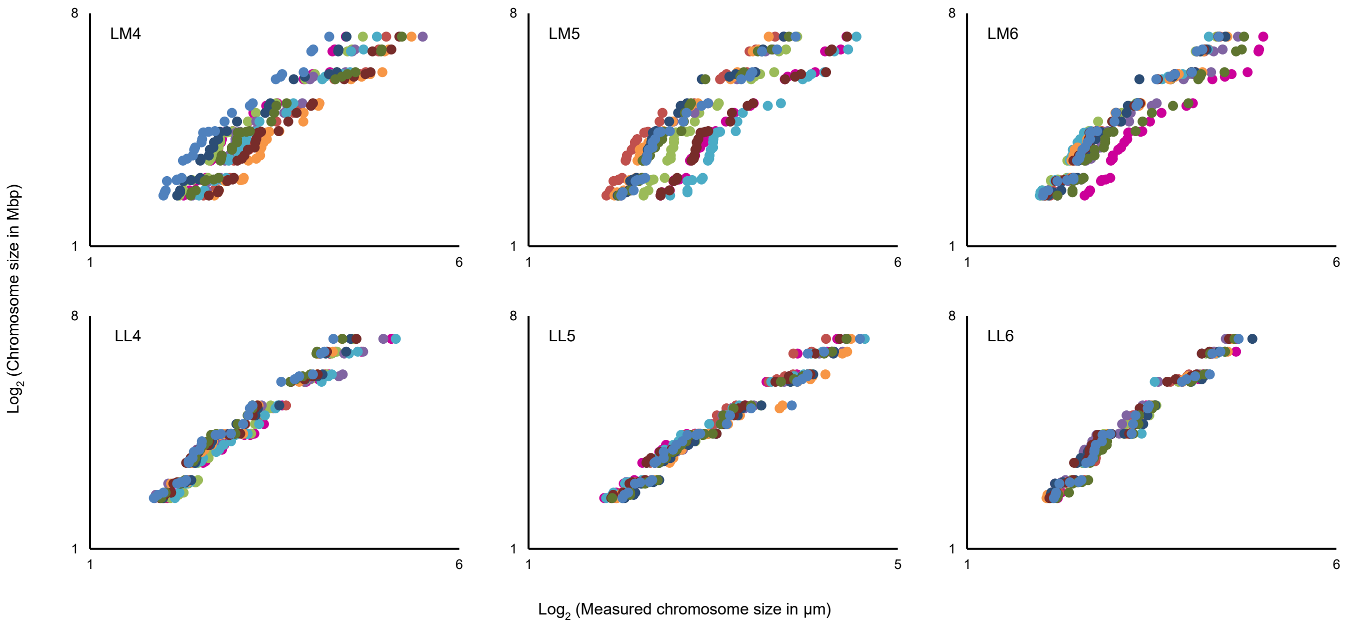

Supplementary Figure 2: Relationship between the measured size and size in bp of the 29 largest chromosomes in 10 cells from 6 individuals in two different species. There is a linear relationship between the logarithmic size of a chromosome's synaptonemal complex (µm) and its logarithmic length in bp (based off *F. albicollis* chromosome sizes). This relationship is consistent within a cell, but not necessarily between cells (each cell is a different colour). This can be seen more clearly in *L. megarhynchos* (top; LM4, LM5, LM6) than in *L. luscinia* (bottom; LL4, LL5, LL6). Source data are provided as a Source Data file.

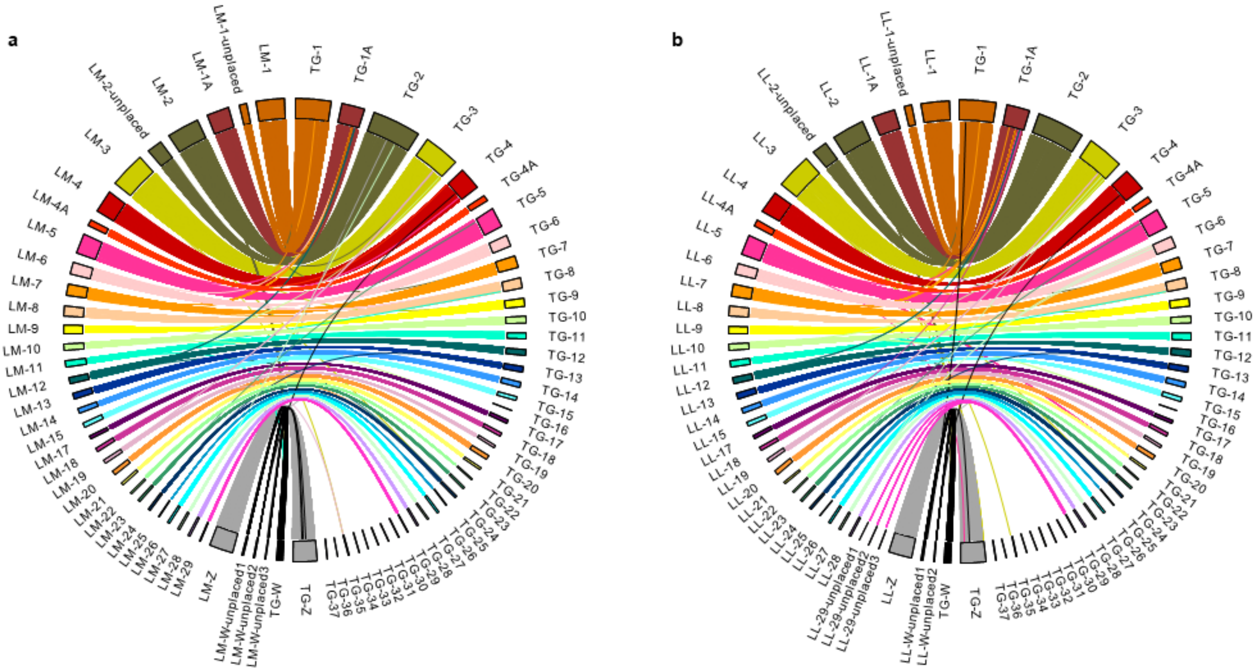

Supplementary Figure 3: Alignment of the nightingale reference somatic genome assemblies to the *T. guttata* genome. Only scaffolds with unambiguous alignments from *L. megarhynchos* (**a**) and *L. luscinia* (**b**) were used, with the exception of the W chromosome and any alignments shorter than 10kb were excluded to improve clarity. The *T. guttata* genome used can be found with the GenBank accession number GCA_003957565.2.

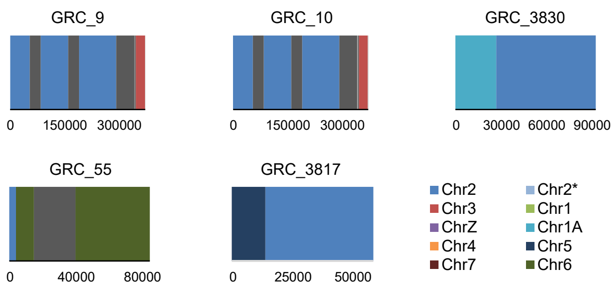

Supplementary Figure 4: Chromosomal origins of select GRC scaffolds from the *L. luscinia* assembly. The shown GRC sequences that are derived from chromosome 2 and are spread across the scaffolds probably originated from a single translocation event, even though they are now often flanked by sequences originating from other chromosomes. Source data are provided as a Source Data file.

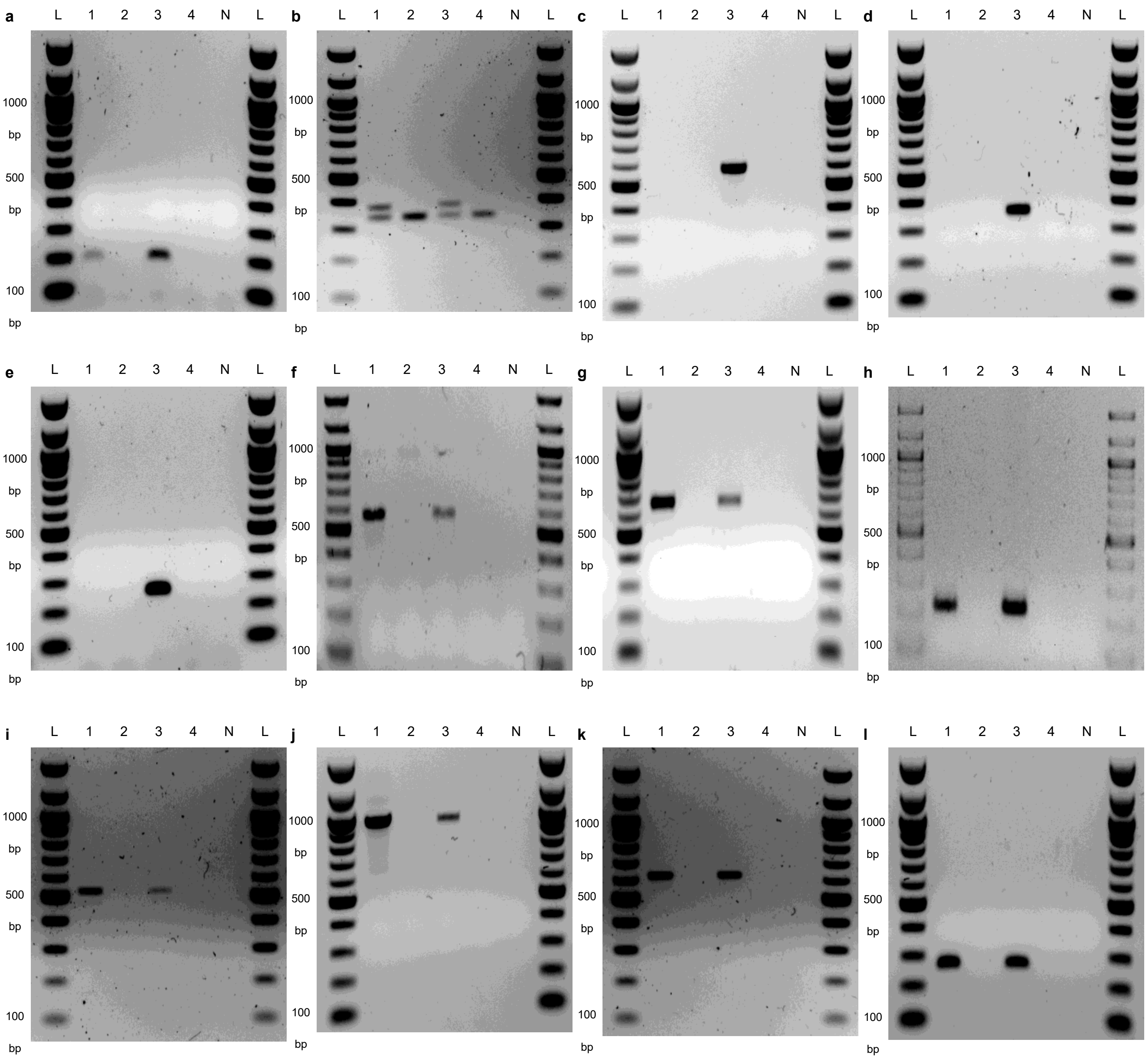

Supplementary Figure 5: PCR products of the GRC validations visualized on 1% agarose gel. The lanes correspond to 100bp New England Biolabs ladder (L), *L. megarhynchos* testis DNA sample (1), *L. megarhynchos* kidney DNA sample (2), *L. luscinina* testis DNA sample (3), *L. luscinina* kidney DNA sample (4), and negative control (N). The primer pairs used were as follows: CPEB1_div_F and CPEB1_div_R (**a**), CPEB1_con_F and CPEB1_con_R (**b**), Chr2_chr6_ext_F and Chr2_chr6_ext_R (**c**), Chr2_chr6_jun_F and Chr2_chr6_ext_R (**d**), Chr2_chr6_ext_F and Chr2_chr6_jun_R (**e**), XP_005051812_F_P1A and XP_005051812_R_P1A (**f**), XP_005046862_F_P2B and

XP_005046862_R_P2C (**g**), XP_016160863_F_P2B and XP_016160863_R_P2A (**h**), XP_005046862_p1_F and XP_005046862_p1_R (**i**), XP_005046862_p2_F and XP_005046862_p2_R (**j**), XP_016160863_p1_F and XP_016160863_p1_R (**k**), and XP_016160863_p2_F and XP_016160863_p2_R (**l**).**b**, **f**, **g** and **h** were also reproduced in a second individual from each species (data not shown).

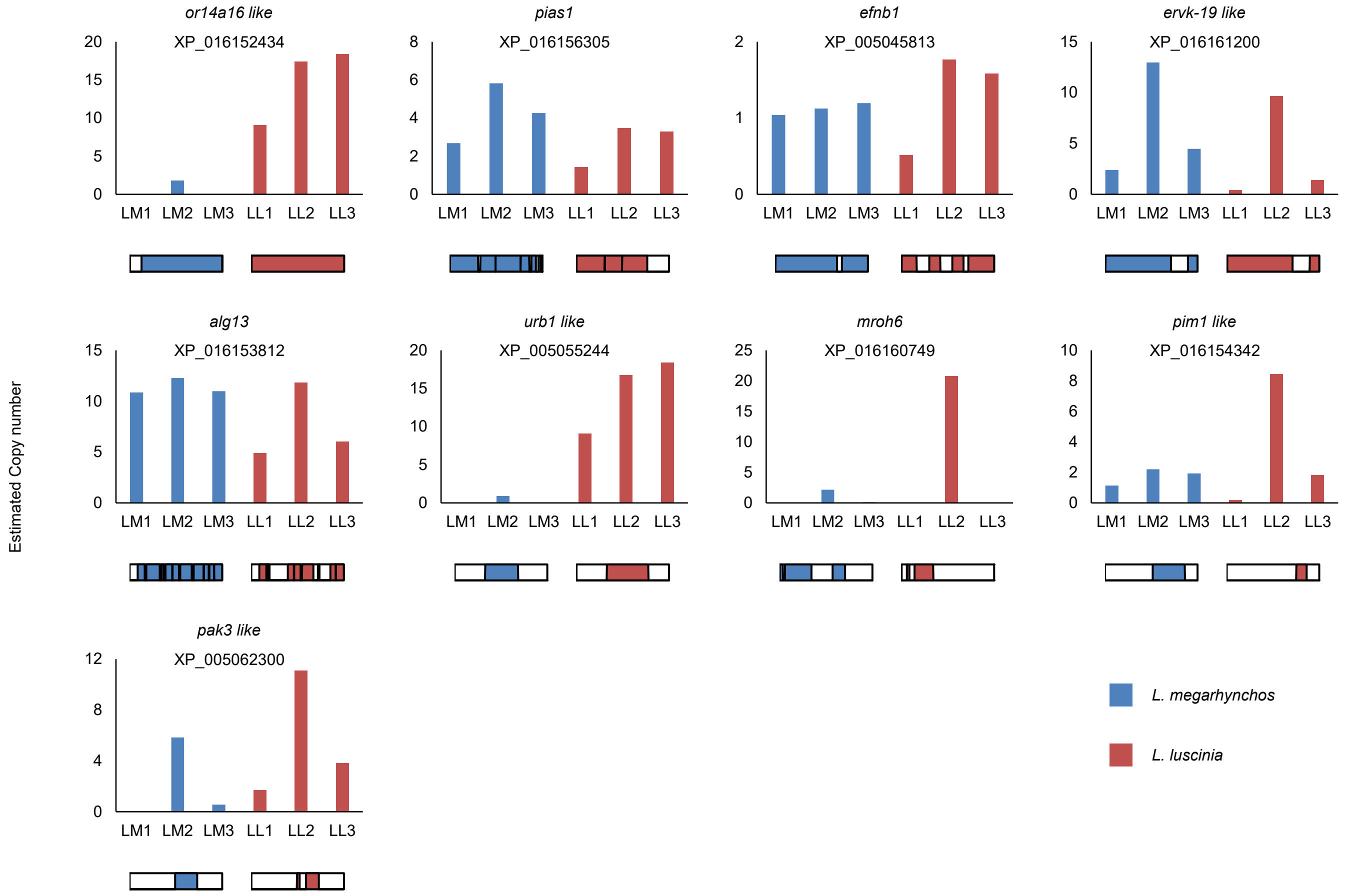

Supplementary Figure 6: Gene fragments shared between the two nightingale species' GRCs and *T. guttata's* GRC. The copy number estimate for each individual is based off the average normalised coverage of each scaffold that the genes were present on. The horizontal bars below the x-axes show the regions of the gene which were found in *L. megarhynchos* (blue) and *L. luscinia* (red). Source data are provided as a Source Data file.

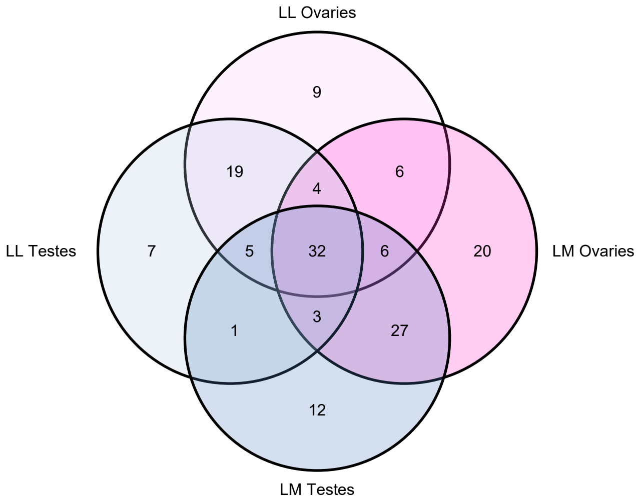

Supplementary Figure 7: Venn diagram showing the number of genes expressed between the different tissues and species. Overall, both tissues had a similar number of genes expressed, with the ovaries (pink) having slightly more expressed genes than the testes (blue). More genes were expressed in *L. megarhynchos* (darker colour and abbreviated to LM) than in *L. luscinia* (lighter colour and abbreviated to LL), although not as a percentage of the genes in their GRC.

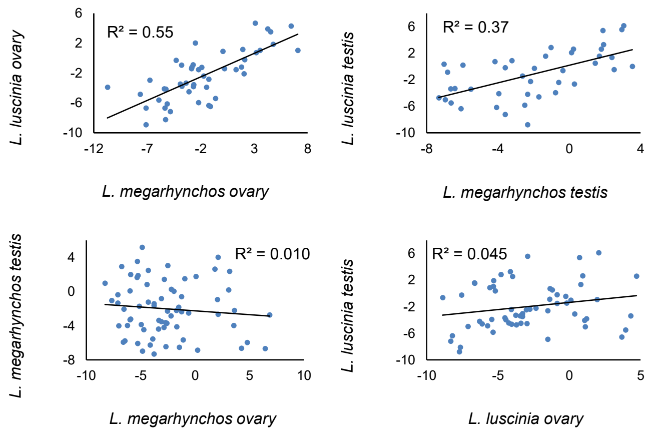

Supplementary Figure 8: Correlation in GRC gene expression between species within tissues and within species between tissues. The between species within tissues comparisons (top) correlate and the within species between tissues comparisons don't (bottom). Ovaries have the highest correlation. Values represent the logarithmic FPKM expression values (base 2). Source data are provided as a Source Data file.

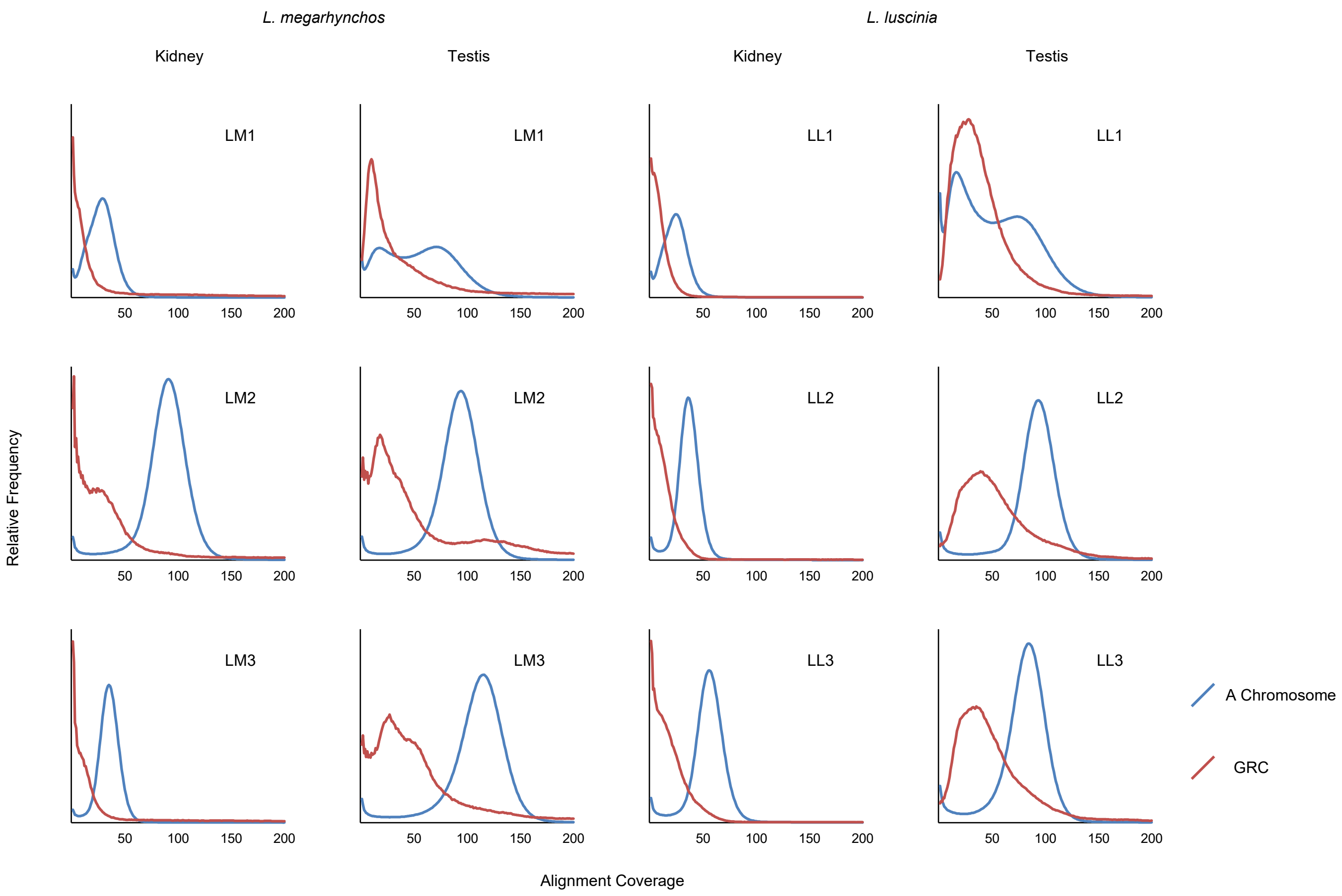

Supplementary Figure 9: Histograms comparing the coverage distributions each sample has over the A chromosomes and GRC. Each individual (abbreviated to LM or LL for *L. megarhynchos* and *L. luscinia*) has two samples associated with it, corresponding to either the kidneys or the testes. Both samples were aligned to the genome and GRC assemblies combined, with the coverage over the A chromosomes (blue) shown separately to the coverage across the GRC (red). The frequency has been normalised to make the GRC comparable to the A chromosomes. Source data are provided as a Source Data file.
