# Supplementary material for: Rapid gene content turnover on the germline-restricted chromosome in songbirds
Source: Nat Commun. 2023 Jul 29;14:4579. doi: 10.1038/s41467-023-40308-8 (PMC10387091; doi:10.1038/s41467-023-40308-8)
Supplement: Supplementary file 3 — Description of Additional Supplementary Files [file 41467_2023_40308_MOESM3_ESM.pdf]

Description of Additional Supplementary Files

File Name: Supplementary Data 1
Description: Estiamtes of GRC size from meitoic spreads for 6 nightinagle individuals. Each estimates is based off a linear regression of chromosome lengths. The average R2 value represents the average of all 10 cells linear regressions.

File Name: Supplementary Data 2
Description: Reference somatic genome statistics for L. megarhynchos and L. luscinia

File Name: Supplementary Data 3
Description: All 192 identified genes from nightingale GRCs. "Bp Found" refers to the number of different nucleotides identified from said gene across all scaffolds. "Adjusted Proportion" represents how much of the cds length was found in the GRC, divided by the proportion of the cds that was found in the whole genome (to a maximum of 0.75). "Normalised Coverage" is the estimated copy number in the GRC, taking recent duplications into account by normalising to the expected coverage using the respective somatic dataset. Expression data is expressed in FPKM, using the length of the gene in the GRC. Note that comparisons of FPKM between datasets is not advised since it is not known what proportion of sequenced cells conatained a GRC.

File Name: Supplementary Data 4
Description: Sequencing depth across 12 libraries. LL represents L. luscinia and LM represents L. megarhynchos. "t" stands for testis and "k" stands for kidney.

File Name: Supplementary Data 5
Description: Description of PCR primers used to validate GRC sequences
