# Supplementary material for: Rapid gene content turnover on the germline-restricted chromosome in songbirds
Source: Nat Commun. 2023 Jul 29;14:4579. doi: 10.1038/s41467-023-40308-8 (PMC10387091; doi:10.1038/s41467-023-40308-8)
Supplement: Supplementary file 5 — Reporting Summary [file 41467_2023_40308_MOESM5_ESM.pdf]

Corresponding author(s): Stephen A. Schlebusch
Radka Reifova

Last updated by author(s): Jul 5, 2023

Reporting Summary

Nature Portfolio wishes to improve the reproducibility of the work that we publish. This form provides structure for consistency and transparency in reporting. For further information on Nature Portfolio policies, see our Editorial Policies and the Editorial Policy Checklist.

Statistics

For all statistical analyses, confirm that the following items are present in the figure legend, table legend, main text, or Methods section.

n/a	Confirmed	
☐	☒	The exact sample size (*n*) for each experimental group/condition, given as a discrete number and unit of measurement
☐	☒	A statement on whether measurements were taken from distinct samples or whether the same sample was measured repeatedly
☐	☒	The statistical test(s) used AND whether they are one- or two-sided<br>*Only common tests should be described solely by name; describe more complex techniques in the Methods section.*
☐	☒	A description of all covariates tested
☒	☐	A description of any assumptions or corrections, such as tests of normality and adjustment for multiple comparisons
☐	☒	A full description of the statistical parameters including central tendency (e.g. means) or other basic estimates (e.g. regression coefficient) AND variation (e.g. standard deviation) or associated estimates of uncertainty (e.g. confidence intervals)
☐	☒	For null hypothesis testing, the test statistic (e.g. *F*, *t*, *r*) with confidence intervals, effect sizes, degrees of freedom and *P* value noted<br>*Give P values as exact values whenever suitable.*
☒	☐	For Bayesian analysis, information on the choice of priors and Markov chain Monte Carlo settings
☒	☐	For hierarchical and complex designs, identification of the appropriate level for tests and full reporting of outcomes
☒	☐	Estimates of effect sizes (e.g. Cohen's *d*, Pearson's *r*), indicating how they were calculated

Our web collection on statistics for biologists contains articles on many of the points above.

Software and code

Policy information about availability of computer code

Data collection	No software was used.
Data analysis	ImageJ v1.50i, Porechop v0.2.4, Flye v2.7.1, Minimap2 v2.17, Nanopolish v0.13.11, Pilon v1.23, Tigmint v1.1.2, Arcs v1.1.1, LINKS v1.8.6, HiRise v0.75, BUSCO v4.0.6, Circos v0.69-9, Fastuniq v1.1, Supernova v2.1.1, Long Ranger v2.2.2, Trimmomatic v0.39, BWA v0.7.17, Samtools v1.14 , GATK v4.1.7.0, KAT v2.4.2, RepeatExplorer2, Blastn v2.10.0, Tblastx v2.10.0, ORFfinder v0.4.3, STAR v2.7.9a, Bedtools v2.27.1, Photoshop v.21.0.2, MegaX v10.2.6 and embedded ClustalW

For manuscripts utilizing custom algorithms or software that are central to the research but not yet described in published literature, software must be made available to editors and reviewers. We strongly encourage code deposition in a community repository (e.g. GitHub). See the Nature Portfolio guidelines for submitting code & software for further information.

Data

Policy information about availability of data

All manuscripts must include a data availability statement. This statement should provide the following information, where applicable:

- Accession codes, unique identifiers, or web links for publicly available datasets
- A description of any restrictions on data availability
- For clinical datasets or third party data, please ensure that the statement adheres to our policy

The novel whole genome Illumina sequencing data generated in this study have been deposited in the NCBI's SRA database under the BioProject accession code

PRJNA808609 [https://www.ncbi.nlm.nih.gov/bioproject/PRJNA808609]. The GRC assemblies have been uploaded onto Figshare (doi: 10.6084/m9.figshare.19161545) and NCBI under the BioProject accession code PRJNA808609 [https://www.ncbi.nlm.nih.gov/bioproject/PRJNA808609] (accessions JAOYSO000000000 and JAOYSP000000000). The assembled reference somatic genomes and their corresponding raw data have been submitted to NCBI (BioProject numbers PRJNA810511 [https://www.ncbi.nlm.nih.gov/bioproject/PRJNA810511] and PRJNA810515 [https://www.ncbi.nlm.nih.gov/bioproject/PRJNA810515]). RNAseq data have been deposited onto NCBI via GEO (ref GSE215907 [https://www.ncbi.nlm.nih.gov/geo/query/acc.cgi?acc=GSE215907]). Source data are provided with this paper. The FicAlb1.5 genomic data used in this study are available in the NCBI database under accession code GCF_000247815.1 [https://www.ncbi.nlm.nih.gov/datasets/genome/GCF_000247815.1/] and the bTaeGut1.4.pri genomic data is found under the accession code GCA_003957565.2 [https://www.ncbi.nlm.nih.gov/datasets/genome/GCF_003957565.2/].

Research involving human participants, their data, or biological material

Policy information about studies with human participants or human data. See also policy information about sex, gender (identity/presentation), and sexual orientation and race, ethnicity and racism.

Reporting on sex and gender	N/A
Reporting on race, ethnicity, or other socially relevant groupings	N/A
Population characteristics	N/A
Recruitment	N/A
Ethics oversight	N/A

Note that full information on the approval of the study protocol must also be provided in the manuscript.

Field-specific reporting

Please select the one below that is the best fit for your research. If you are not sure, read the appropriate sections before making your selection.

☐ Life sciences ☐ Behavioural & social sciences ☒ Ecological, evolutionary & environmental sciences

For a reference copy of the document with all sections, see nature.com/documents/nr-reporting-summary-flat.pdf

Ecological, evolutionary & environmental sciences study design

All studies must disclose on these points even when the disclosure is negative.

Study description	The germline restricted chromosome from two nightingale species was assembled using sequencing data from 3 individuals per species. This was done by comparing the sequencing data from somatic (kidney) and germline (testis) tissue from each individual.
Research sample	This study uses two closely related songbird species (Luscinia megarhynchos and Luscinia luscinia). We chose closely related songbird species because the GRC has shown large variability over short evolutionary time spans. We specifically chose nightingales because our research team has experience working with them. The whole genome sequencing used males from each species because the testes have a lot more germline cells in them than ovaries. Females were only used for ovary RNAseq.
Sampling strategy	Random wild individuals were sampled. No sample size calculation was performed. Three individuals were chosen for whole genome germline sequencing from each species as it gives an impression of within species sequence variance while still allowing for sufficient sequencing coverage of each individual. Three individuals were were used for meiotic spread visualisations as it was the minimum amount we would have to kill and still be able to estimate varaince. Only 1 male and female were used for RNAseq as we were mostly interested in the presence or absence of expression of identified genes.
Data collection	DNA was sequenced using long-read Nanopore sequencing, standard Illumina sequencing, 10x Genomics linked-read sequencing and Omni-C chromatin conformation capture. RNA was sequenced using Illumina sequencing.
Timing and spatial scale	Samples were collected during their breeding season in the first half of May in 2018 and 2019.
Data exclusions	No data was excluded from the study.
Reproducibility	We prepared 12 PCR primer pairs to amplify 5 different regions and confirm that the assembled sequences were genuinely unique to the germline, including the most important gene from the study (cpeb1), as well as the largest source of differentiation between the species, a large duplication from chromosome 2. Four of these primer pairs were additionally tested in a second individual from each species to confirm that the results were consistent.
Randomization	Samples could not be randomised in this study. Samples needed to be matched with their respective species and tissue.
Blinding	It was necessary to know which sample belonged to which species and which tissue for effective analysis and so no blinding was

Blinding	possible.

Did the study involve field work? ☒ Yes ☐ No

Field work, collection and transport

Field conditions	All samples were collected in allopatric regions near rivers where they tend to live.
Location	South-western Poland, near the town of Brzeg Dolny, by the Odra River (N 51°26′02′′, E 16°74′40′′) and North-Eastern Poland, near the town Łomża, by the Narew Rriver (N 53°16′21′′, E 22°12′46′′).
Access & import/export	This work was carried out in accordance with ethical animal research requirements of Poland according to Polish law (the Act On the Protection of Animals used for Scientific or Educational Purposes, 15.01.2015, item 266, implementing Directive 2010/63/EU of the European Parliament and of the European Council of 22.09.2010).
Disturbance	Researchers made an effort to keep noise to a minimum.

Reporting for specific materials, systems and methods

We require information from authors about some types of materials, experimental systems and methods used in many studies. Here, indicate whether each material, system or method listed is relevant to your study. If you are not sure if a list item applies to your research, read the appropriate section before selecting a response.

Materials & experimental systems

n/a	Involved in the study
☐	☒ Antibodies
☒	☐ Eukaryotic cell lines
☒	☐ Palaeontology and archaeology
☐	☒ Animals and other organisms
☒	☐ Clinical data
☒	☐ Dual use research of concern
☒	☐ Plants

Methods

n/a	Involved in the study
☒	☐ ChIP-seq
☒	☐ Flow cytometry
☒	☐ MRI-based neuroimaging

Antibodies

Antibodies used	Rabbit polyclonal anti-SYCP3 antibody (ab15093; Abcam)(dilution 1:200), Human anticentromere serum (CREST; 15-234; Antibodied Incorporated) (dilution 1:50) , Rabbit monoclonal anti-H3K9me3 antibody (ab8898, Abcam) (dilution 1:200)
Validation	anti-H3K9me3 and CREST are used in Del Priore et al (Del Priore, Lucía, and María Inés Pigozzi. Histone modifications related to chromosome silencing and elimination during male meiosis in Bengalese finch. Chromosoma 123:3: 293-302 (2014))<br><br>anti-SYCP3 is used in Torgasheva et al (Torgasheva, A. A., et al. Germline-restricted chromosome (GRC) is widespread among songbirds. Proc. Natl. Acad. Sci. U. S. A. 116:, 24:, 11845-11850 (2019). doi: 10.1073/pnas.1817373116)

Animals and other research organisms

Policy information about studies involving animals; ARRIVE guidelines recommended for reporting animal research, and Sex and Gender in Research

Laboratory animals	The study did not involve laboratory animals.
Wild animals	Seven adult males of the common nightingale (Luscinia megarhynchos) and the thrush nightingale (L. luscinia) were caught in the field using mist nets. Birds were sacrificed using cervical dislocation. From three male individuals, somatic (kidney) and gonadal (testis) tissues were dissected for DNA isolation and sequencing. Another 3 individuals had testes isolated for microscopy. The final male had testes isolated for mRNA sequencing. Additionally, 1 adult female from each species was caught and sacrificed sing cervical dislocation. This bird was used for Nanopore and Omni-C sequencing as well as mRNA sequencing from its ovaries.
Reporting on sex	The study compares the expression patterns from ovaries and testes in the two nightingale species.
Field-collected samples	The wild animals described above were collected in the field.
Ethics oversight	The work was approved by the General Directorate for Environmental Protection, Poland (permission no. DZP-

Ethics oversight	WG.6401.03.123.2017.dl.3).

Note that full information on the approval of the study protocol must also be provided in the manuscript.
